# Microscopic optical buffering in a harmonic potential

**DOI:** 10.1038/srep18569

**Published:** 2015-12-22

**Authors:** M. Sumetsky

**Affiliations:** 1 Aston Institute of Photonic Technologies, Aston University, Birmingham B4 7ET, UK

## Abstract

In the early days of quantum mechanics, Schrödinger noticed that oscillations of a wave packet in a one-dimensional harmonic potential well are periodic and, in contrast to those in anharmonic potential wells, do not experience distortion over time. This original idea did not find applications up to now since an exact one-dimensional harmonic resonator does not exist in nature and has not been created artificially. However, an optical pulse propagating in a bottle microresonator (a dielectric cylinder with a nanoscale-high bump of the effective radius) can exactly imitate a quantum wave packet in the harmonic potential. Here, we propose a tuneable microresonator that can trap an optical pulse completely, hold it as long as the material losses permit, and release it without distortion. This result suggests the solution of the long standing problem of creating a microscopic optical buffer, the key element of the future optical signal processing devices.

One of the greatest challenges of the modern photonics is the creation of miniature low-loss and high speed optical signal processors which promise to revolutionize the future computing and communications^1^,^2^. The key and most provocative element of these devices enabling the control and manipulation of optical pulses is the microscopic optical buffer. Having the smallest possible dimensions, the buffer should trap optical pulses, hold them for the required (usually nanosecond-order) period of time, and release them without distortion. The delay of an optical pulse in a small-size photonic structure assumes that the pulse experiences many oscillations (e.g., reflections and rotations) before being released, i.e., its propagation speed averaged over these oscillations is slow. Searching for realistic miniature optical delay lines and buffers based on this “slow light” concept resulted in several remarkable designs employing coupled ring resonators and photonic crystal waveguides^1^,^2^,^3^,^4^,^5^,^6^. The most important of these slow light structures are periodic and their transmission band has a region of approximately zero dispersion, which ensures the nearly dispersionless and slow propagation of optical pulses. These structures though suffer from the bandwidth-delay time limitation, which restricts the values of the delay time and pulse bandwidth achieved simultaneously^1^,^2^,^7^. A way to overcome this limitation suggested in^6^ consists in creation of a miniature optical buffer by adiabatic compression of the transmission band of a coupled resonator optical waveguide. Ideally this device enables slowing down and stopping of a light pulse with the predetermined spectral width. Yet, the experimental realization of all these models encountered significant practical barriers due to insufficient precision of modern photonics technologies and attenuation of light^8^. Consequently, it was suggested that “slow light dispersion, bandwidth, and loss are fundamental issues that will limit the use of slow light devices as buffers”^9^.

However, in 1926, soon after the creation of quantum mechanics, Erwin Schrödinger published a paper where he investigated the oscillations of a Gaussian wave packet (used as a model of a quantum particle) in a harmonic potential^10^. He showed that the particle oscillations are periodic, i.e., the wave packet in a harmonic potential does not experience any distortion after many oscillations. Schrödinger wrote: “*Our wave group always remains compact,* and does *not* spread out into larger regions as time goes on, as we were accustomed to make it do, for example, in optics. It is admitted that this does not mean much in one dimension…” While the quantum motion of wave packets in a one-dimensional harmonic potential is indeed difficult to find in nature, the wave packet dynamics in potential wells with more general anharmonic shapes and higher dimensions has been investigated both theoretically and experimentally in atomic and solid state physics. It has been found that, unlike in one-dimensional harmonic potential, this motion is generally not periodic, though often exhibits the revival behaviour^11^.

Remarkably, Schrödinger’s result can be obtained without solving the Schrödinger equation for the potential with the quadratic dependence on the coordinate. In fact, it is sufficient to assume that the potential well has an equidistance spectrum 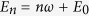 corresponding to the stationary wave functions 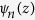. Then, from the linearity of quantum mechanics, the evolution of a wave packet 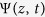 with arbitrary initial shape 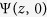 is found as 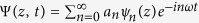 which has the period 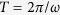. Thus, the periodicity of wave packet oscillations follows from the equidistance of the spectrum of the resonator within the spectral width of the wave packet, which can be ensured by potentials with more general spatial dependencies.

We note that, in photonics, similar resonators (i.e., those having the equidistance spectrum within the pulse spectral width) can serve as ideal miniature optical buffers since they can hold optical pulses without distortion. In contrast to quantum mechanics, where the experimental realization of such resonators is problematic and still does not mean much in one dimension^10^, essentially one-dimensional resonant structures can be realized based on the photonic crystal waveguides, sequences of ring resonators, and fibre Bragg gratings. To this end, the periodicity of these structures should be appropriately chirped^12^,^13^,^14^ to arrive at the locally precise equidistant spectrum. Yet, similar to the above mentioned approaches based on the subwavelength-scale modulation of refractive index, the practical realization of these structures is impeded by insufficient fabrication precision and substantial attenuation of light^3^,^8^,^9^.

On the other hand, the recently developed photonic fabrication platform, Surface Nanoscale Axial Photonics (SNAP), precisely imitates the one-dimensional Schrödinger equation optically and, at the same time, does not require the subwavelength-scale modulation of the refractive index to arrive at the effective potential with required spectrum governing the slow light propagation^15^,^16^,^17^. The photonic structures in SNAP are created at the surface of an exceptionally smooth and uniform optical fibre by its nanoscale deformation with the unprecedented subangstrom precision. Instead of periodicity, which warrants the slow light propagation in photonic crystals, this platform explores whispering gallery modes, which experience multiple transverse circulations and slowly propagate along the fibre axis. In particular, a SNAP bottle resonator^15^,^16^,^17^ with the parabolic effective radius variation can accurately reproduce the Schrödinger’s harmonic potential well. The parabolic bottle resonator delay line experimentally demonstrated in^17^ was introduced with a subangstrom precision. The resonator was fabricated at the 3 mm segment of an optical fibre with 19 μm radius, i.e., had the footprint of 0.12 mm^2^. The effective radius variation of this resonator had the 2.8 nm height, which allowed us to delay 100 ps optical pulses by 2.5 ns. To avoid the reflection of an optical pulse exiting the resonator, coupling between the bottle resonator and the input-output microfibre was tuned to satisfy the impedance matching condition. As the result, the record small insertion loss of a slow light delay line in excess of 0.5 dB/ns was demonstrated.

Here, we propose a miniature bottle resonator optical buffer based on the generalised tuneable harmonic oscillator introduced below. This device presents a feasible solution of the long standing problem of creating a smallest possible microscopic buffer for processing of optical pulses. To illustrate the idea of the paper, we first compare oscillations of an optical pulse launched into the stationary harmonic resonator, which exhibits no distortion, and anharmonic (rectangular) bottle resonator, which exhibit dramatic distortion. Next, we construct a non-stationary potential corresponding to a tuneable bottle resonator and show that it can work as a perfect optical buffer, i.e., it can trap an optical pulse, hold it for a predetermined period of time, and release it without distortion. Then, we take into account fabrication errors and show that the required fabrication precision is achievable. In the final Discussion section, the feasibility of the proposed harmonic optical buffers is analysed based on the recent progress in fabrication and investigation of nonlinear and piezoelectric multimaterial optical fibres.

## Results

### Optical pulse oscillations in a potential well

Let us first establish the correspondence between the Schrödinger equation, which describes the motion of a one-dimensional quantum particle^18^, and the Schrödinger equation, which describes propagation of light in the SNAP platform. Due to the very small and smooth effective radius variation of a SNAP structure, a whispering gallery mode (WGM) can be determined by separation of variables in cylindrical coordinates 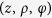 as 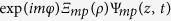. Below we consider the resonant propagation of a WGM pulse corresponding to the fixed azimuthal and radial quantum numbers 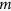 and 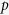, which is fully described by the amplitude 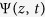 as a function of axial coordinate 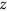 and time 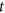 (here and below the quantum number indexes are omitted for brevity). The equation that determines this propagation has the form of the one-dimensional Schrödinger equation^15^, which for the non-stationary case under consideration takes the form (see Methods):


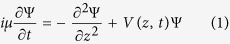


Here 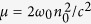 and potential 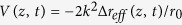 are defined through the radiation frequency 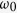 of the transmission channel, refractive index 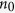 and bulk propagation constant 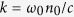 of the bottle resonator material, speed of light in vacuum 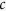, and fibre radius 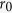. The nanoscale effective variation 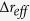 of the fibre radius is expressed through the variation of the effective physical radius 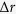 and refractive index 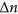 as 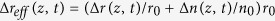
15. Experimental results^16^,^17^ demonstrate the fabrication of SNAP structures with sub-angstrom precision in effective radius variation, while the fabrication precision of 0.1 angstrom is also feasible.

**Figure 1:**
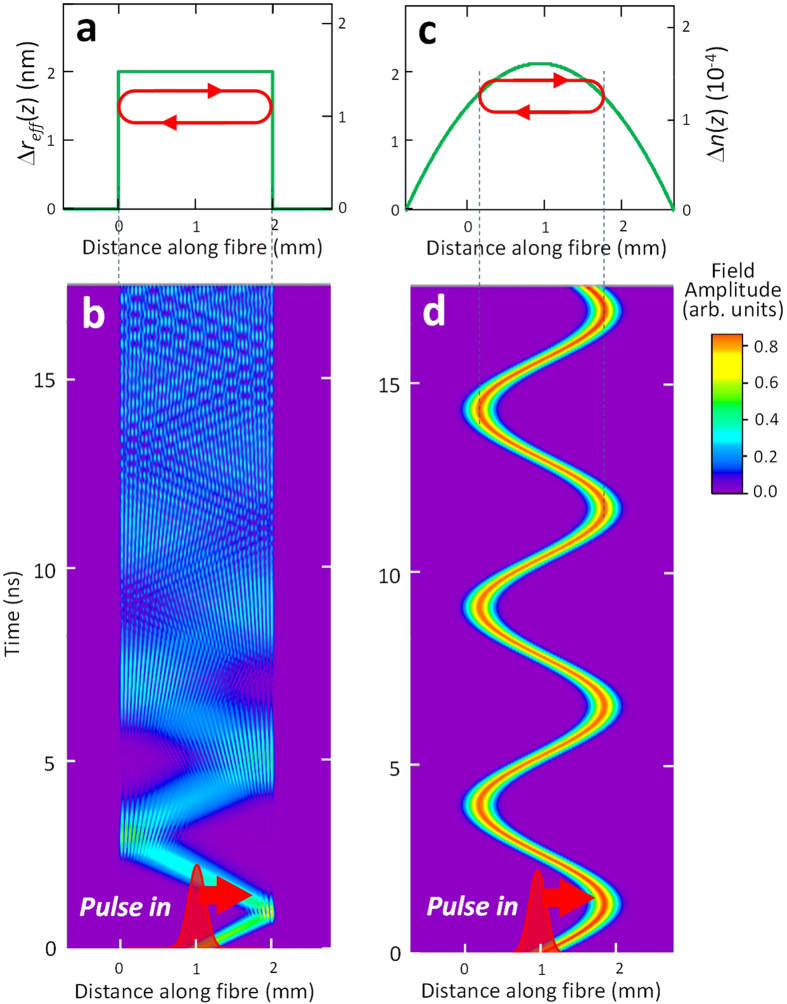
Propagation of a 100 ps optical pulse in rectangular and parabolic bottle resonators. **(a)** Effective radius variation of the rectangular resonator having 2 nm height and 2 mm length. **(b)** Propagation of a 100 ps optical pulse in this resonator. The surface plot shows the field distribution of the pulse as a function of the coordinate along the fibre and time. It is seen that the pulse is completely corrupted after several nanoseconds of propagation and a few reflections due to the dispersion and self-interference. **(c)** Effective radius variation of the parabolic resonator having the 345 m radius of curvature. **(d)** Propagation of a 100 ps pulse in this resonator showing the periodic oscillations of the pulse with no distortion over the oscillation cycles.

To illustrate the effect of dispersion and self-interference, we first consider the propagation of an optical pulse launched into the *rectangular* bottle resonator having the height 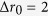
nm and length 2 mm (Fig. 1a). Here and below, we consider the propagation of 100 ps pulses and set the fibre radius 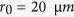, refractive index 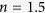, and radiation wavelength 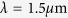. The spatial width of the 100 ps Gaussian pulse is 4.2 cm in vacuum and approximately 2.8 cm in a silica fibre. Following the experimental observations^17^, we set the initial *axial* speed of the pulse to the realistic 1% of its actual speed in silica, i.e., to 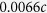. Consequently, the axial width of the pulse is reduced to 0.28 mm. The surface plot describing the evolution of this pulse along the fibre axis 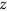 as a function of time is shown in Fig. 1b. It is seen that the pulse experiences significant corruption in the process of bouncing caused by both the dispersion and self-interference^11^,^19^. As the result, the original shape of the pulse is completely lost in a few nanoseconds. In contrast, as was first noted by Schrödinger^10^, oscillations of an optical pulse in a bottle resonator with quadratic radius variation profile 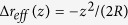 and axial radius 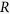 are periodic and do not cause dispersion over time as illustrated in Fig. 1c,d.

### Harmonic optical buffer

**Figure 2:**
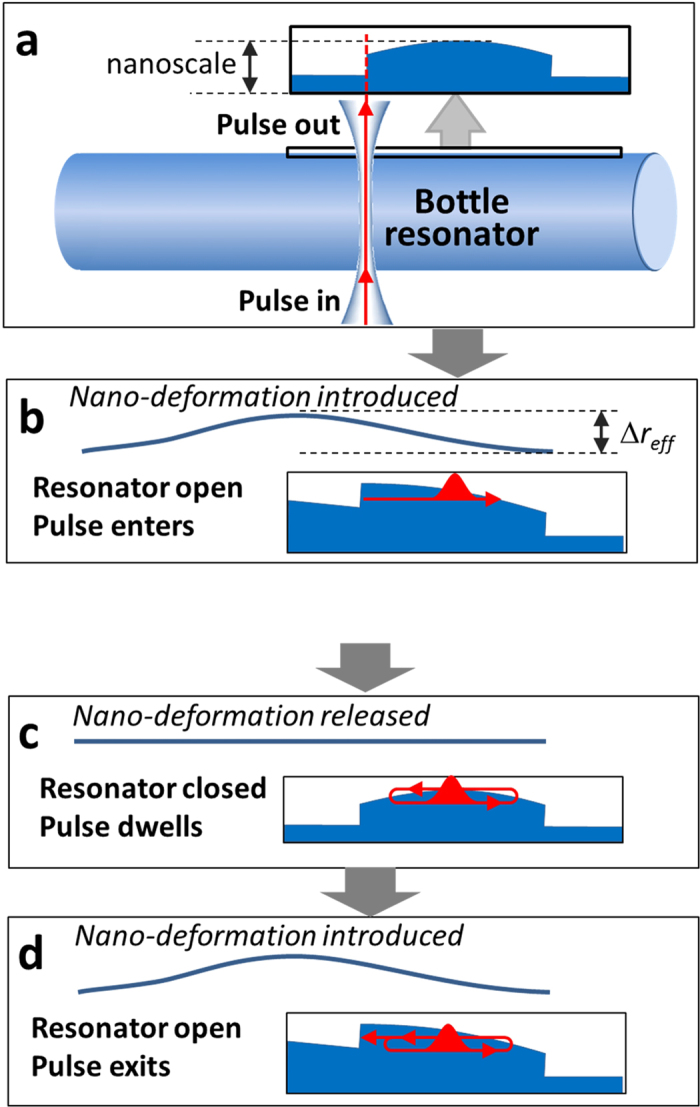
Illustration of a SNAP bottle resonator optical buffer. (**a**) A SNAP bottle resonator created from an optical fibre with nanoscale parabolic radius variation. The resonator is coupled to the transverse input-output waveguide (micrometer-diameter waist of an optical fibre taper). **(b)** The switching nano-deformation of the effective radius, which is introduced by a pulse of the applied laser or electrical field, transfers the closed parabolic resonator into the open semi-parabolic resonator shown in this figure. (**c**) After the deformation shown in Figure (**b**) is released, the bottle resonator restores its original parabolic shape with the optical pulse oscillating inside it. **(d)** Finally, the same as in Figure (**b**) nano-deformation is introduced again and the pulse is released back into the input-output waveguide. The red curves illustrate the propagation of an optical pulse in each of the configuration considered.

Generalizing the Schrödinger’s result^10^, we introduce now a harmonic optical buffer. We show that a tuneable harmonic potential well, which is reproduced by a miniature SNAP bottle resonator illustrated in Fig. 2, can trap an optical pulse completely, hold it as long as the material losses permit, and release without distortion. Light is coupled in and out of this resonator through a transverse waveguide, e.g., a microfibre taper (Fig. 2a). The buffering process includes: opening the bottle resonator by nanoscale variation of its effective radius (refractive index) to let the optical pulse in (Fig. 2b); closing the resonator when the pulse is completely inside the resonator and holding it for the duration of the required time delay (Fig. 2c); and releasing the pulse by reversing the deformation illustrated in Fig. 2b (Fig. 2d). The pulse dwell time in the optical buffer is not restricted by the delay time-bandwidth limitation since it is determined only by the number of oscillation cycles and material losses which allow the pulse to oscillate in the buffer without significant attenuation. Feasible experimental ways to open and close the bottle resonator by the application of laser and electrical pulses will be described later.

The bandwidth 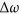 of the optical pulse that can be held in the bottle resonator is expressed through the magnitude of its effective radius variation 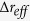 as


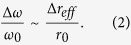


For the case of purely refractive index tuning, this equation coincides with 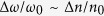
7,20. The amplitude of the effective radius variation required for opening and closing the resonator (Fig. 2b) is determined from equation (2) as well. For a 100 ps Gaussian pulse, which at telecommunication wavelength 1.5 μm (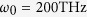) has the spectral width 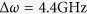, equation (2) yields 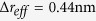. Thus, a nanometre-shallow bottle resonator can fully confine 100 ps optical pulses, while opening and closing the resonator requires just a nanometre-high variation of the effective fibre radius.

**Figure 3:**
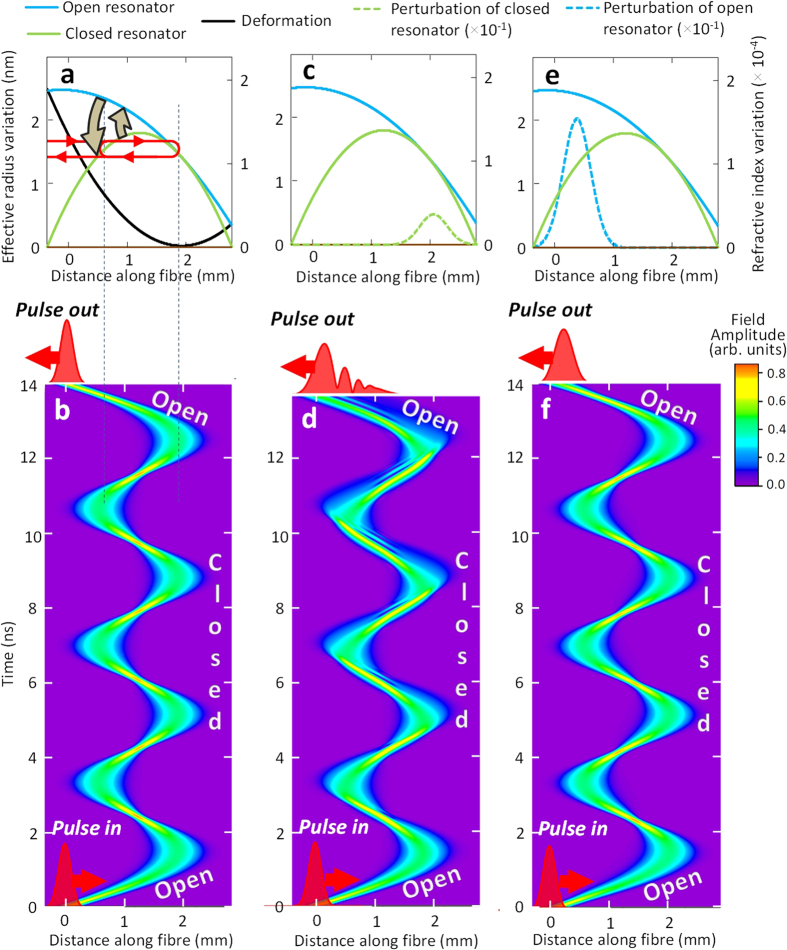
Performance of a SNAP bottle resonator optical buffer. (**a**) The effective radius variation (left vertical axis) and equivalent refractive index variation (right vertical axis) of the open semi-parabolic resonator (blue curve) and closed parabolic resonator (green curve). The nano-deformation (black curve), equal to the difference of these curves, is gradually introduced and released during a sub-nanosecond time period. **(b)** The surface plot shows the distribution of the field of the optical pulse, which is captured, held, and released by the buffer, as a function of the coordinate along the bottle resonator and time. The output pulse shown at the top of the figure (and also shown in Fig. 4b) exhibits the negligible distortion compared to the input pulse at the bottom. **(c)** In this figure, the closed parabolic resonator shown in **(a)** is perturbed by a Gaussian deformation with the height of 0.5 Å (dashed green curve). **(d)** The surface plot in this figure shows that the optical buffer with such perturbed radius variation exhibits significant distortion of the pulse over time (the output pulse for this case is also shown in Fig. 4e). (e) This figure shows a much greater 2 Å Gaussian perturbation with the same width introduced at the entrance of the open semi-parabolic resonator (dashed blue curve). **(f)** The evolution of the pulse in the optical buffer with such perturbation exhibits the tolerable distortion of the pulse (shown at the top of (**f**) and also in Fig. 4g).

As opposed to adiabatically slow tuning, which, ideally, is reversible and therefore allows acquiring and releasing a pulse without distortion^6^, we do not assume here that the switching process is slow. Instead, we show that it is possible to introduce a fast deformation (refractive index variation) of the bottle resonator without disturbing (i) the optical pulse shape, (ii) the harmonicity of the potential, and, as a consequence, (iii) the reversibility of the buffering process. To arrive at such microscopic optical buffer, the time-dependent potential 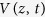 is constructed as follows. First, we request that, when fully opened, this potential coincides with a harmonic semi-parabolic potential


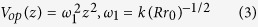


proportional to the effective radius variation of the bottle resonator 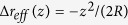 with axial radius 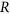 (Fig. 3a,b, blue curves). Next, we look for the parabolic closed potential centred at point 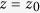 in the form


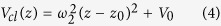


and choose 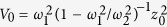 so that these potentials are tangent at their common point 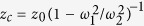 (Fig. 3a,b, dashed red curves). Finally, the time-dependent buffering potential 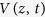 is constructed as 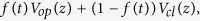 where the switching function 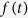 is equal to zero and one for the closed and open potential, respectively. The switching process will not disturb the optical pulse significantly if the characteristic switching time is less than the time it takes the pulse to reside in the left hand side region of 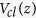 near the common point 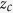 where potentials 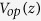 and 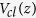 are approximately equal.

As an example, we numerically investigate a miniature optical buffer having the open and closed configurations defined by the harmonic semi-parabolic and parabolic effective radius variations shown in Fig. 3a. Buffering of a 100 ps Gaussian pulse is shown in the surface plot of Fig. 3b. The bottle resonator captures the pulse between the 1^st^ and 2^nd^ ns after launch, holds it over the duration of 4 oscillation cycles and releases with practically no distortion between the 12^th^ and 13^th^ ns. The total delay time is 14 ns, while the time of one cycle is 3.6 ns. Comparison of Fig. 3a,b shows that, in agreement with equation (2), the pulse is fully confined by a parabolic resonator which is as shallow as 1 nm in effective radius variation.

### Optical buffering in a generalized harmonic potential

The parabolic potential is not the only one that possesses the equidistant spectrum and supports the periodic oscillations similar to those shown in Fig. 1d. Here we show that a wide class of non-parabolic potentials support the periodic oscillations of an optical pulse with excellent accuracy. This result is critical for practical realization of the proposed optical buffer since it makes its design much more flexible.

We assume here that the potential well is wide enough to ensure a relatively large oscillation time and high enough to ensure the sufficient bandwidth of the pulse. Thus, we are interested in potentials which can be treated semi-classically^18^. It is shown in the Supplementary Note that the family of potentials 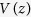 which are harmonic in this approximation are determined by the algebraic equation for their inverse functions 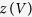:


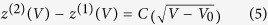


where 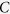 and 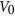 are arbitrary constants. In this equation, one branch of the potential, e.g., 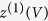, can be an arbitrary monotonic function, while the other branch, 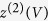, is expressed through 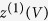 from equation (5).

**Figure 4:**
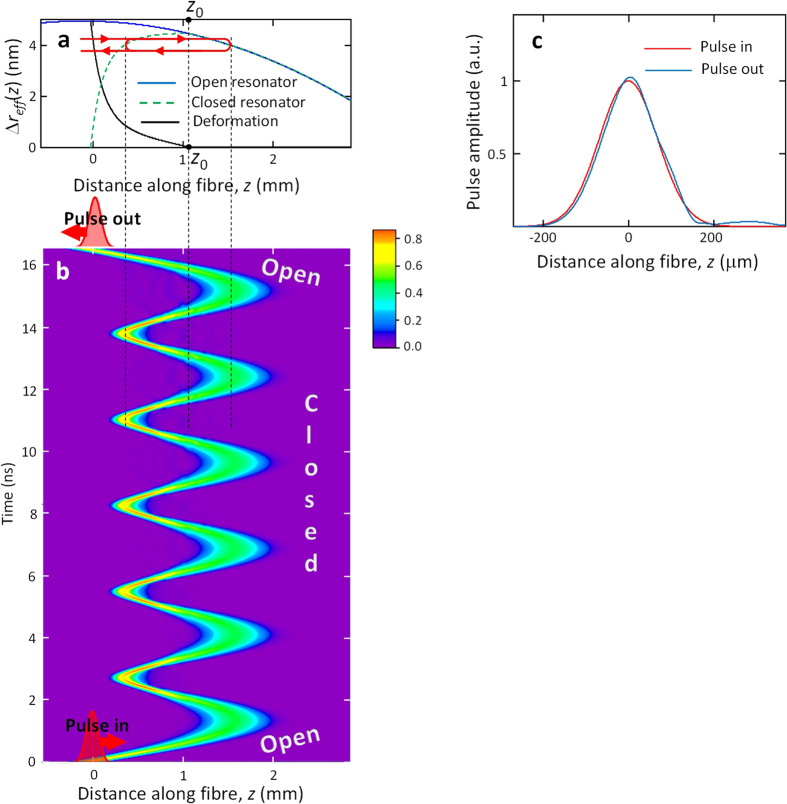
Performance of a SNAP bottle resonator optical buffer constructed of an asymmetric semi-classical harmonic potential. (**a**) The effective radius variation of the open semi-parabolic resonator (blue curve) and closed parabolic resonator (green dashed curve). The nano-deformation (black curve), equal to the difference of these curve, is gradually introduced and released during a sub-nanosecond time period and is equal to zero at *z* > *z*_0_. **(b)** The surface plot shows the distribution of the field of the optical pulse, which is captured, held, and released by the buffer, as a function of the coordinate along the bottle resonator and time. The output pulse shown at the top of the figure exhibits the negligible distortion compared to the input pulse at the bottom. **(c)** Comparison of the input and output pulse profiles for the pulse propagation shown in **(b)**.

Let us construct the buffer potential 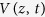 (or, equivalently, the effective radius variation 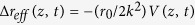) as follows. First, we request that, when fully opened, this potential coincides with a harmonic semi-parabolic potential defined by equation (3) (Fig. 4a, blue curve). In the process of switching, we request that the left hand side region, 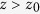, of potential 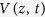, remains unchanged so that the optical pulse propagating in this region at this time is *not perturbed by switching at all*. This is different from the model considered in the previous section where the transition between the closed and open potentials deformed the closed potential and therefore slightly perturbed the optical pulse. Next, we request that, after full closing, the new left hand side of the potential, at 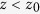, together with the remaining right hand side 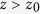 form a closed harmonic potential 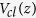 (Fig. 4a, green dashed curve) where the optical pulse can oscillate without distortion. The analytical expression for 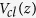, is determined from equation (5) as:


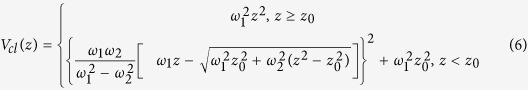


where 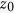, 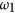, and 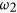 are free parameters. As requested, potential 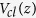 has the equidistant spectrum in the semi-classical approximation. It is asymmetric and, though continuous everywhere, has a break of the first derivative at 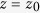. Finally, the time-dependent buffering potential 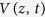 is constructed as 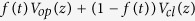 as in the previous section. The switching process will not disturb the pulse if the characteristic switching time is less than the time it takes the pulse to reside in the region 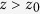.

The buffering process is described as follows. The optical pulse is launched into the open semi-parabolic potential. When the pulse is approaching the right hand side turning point, the semi-parabolic potential is gradually closing and transforming into the asymmetric harmonic potential determined by equation (6) (Fig. 4a). Crucially, this deformation does not affect the right hand side of the potential, which is the *common parabolic part* of both the closed and open harmonic potentials. For this reason, the deformation does not perturb the pulse, which at the time of deformation is situated completely within the right hand side parabolic part of the potential well. For the next period of time, the pulse is oscillating between turning points in the closed potential without distortion. Finally, the inverse time-dependent process transforms the closed parabolic potential into the open semi-parabolic potential to let the pulse out. Again, this process does affect the shape of the optical pulse.

As an example, we numerically investigate a miniature optical buffer having the opened and closed configurations defined by the harmonic semi-parabolic and asymmetric effective radius variations shown in Fig. 4a. Buffering of a 100 ps Gaussian pulse is shown in the surface plot of Fig. 4b. The bottle resonator captures the pulse, holds it over the duration of 6 oscillations and releases with practically no distortion. For the 2 nm high effective radius variation considered, the single oscillation time is 1.7 ns and the whole delay is around 16 ns. The slight discrepancy between the input and output pulses shown in Fig. 4c can be explained by the fact that the closed potential was determined in the semi-classical approximation, i.e., it is not the exact harmonic potential. However, the remarkably small distortion shown in Fig. 4c is obviously sufficient for practical applications. In addition, it is expected that the potential profile can be iterated to minimize the distortion further.

### Required fabrication precision

**Figure 5:**
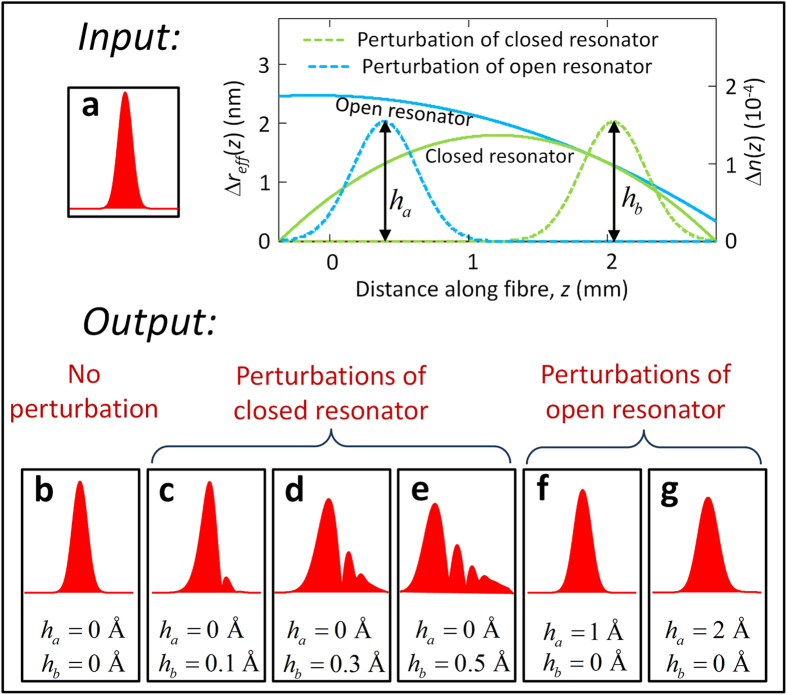
Comparison of the input 100 ps pulse (a) and output pulses corresponding to different perturbations of the optical buffer (b–g). The profiles of 0.6 mm FWHM Gaussian perturbations of the closed parabolic resonator (green dashed curve with height *h*_*a*_) and open semi-parabolic resonator (blue dashed curve with height *h*_*b*_) which are positioned as shown in the inset. (**b**) The output pulse for the unperturbed buffer. (**c**–**e**) The output pulses for the closed resonator perturbations with 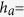
0.1, 0.3, and 0.5 Å, respectively. (**f**,**e**) The output pulses for the open resonator perturbations 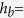
1 and 2 Å, respectively.

Since the absolutely precise harmonic potential cannot be realized experimentally, it is important to determine the fabrication precision required for satisfactory performance of the buffer. We verify the effect of deviation from the harmonicity by adding a perturbation (a) inside the closed harmonic potential, which disturbs the process of periodic oscillations and (b) at the entrance of the open harmonic potential, which affects the process of entering and exiting of the resonator. Calculations show that perturbations of the closed harmonic potential, especially those localized near the turning points of the pulse, have a much stronger effect on the distortion of the optical pulse than those of the open semi-parabolic potential. In fact, the pulse speed near the turning points tends to zero, so that the acquired effect of a perturbation here is maximized. Furthermore, the effect of perturbation of the closed potential is multiplied by the number of oscillation cycles. We assume that the perturbations are spatially smooth and choose them in the form of a Gaussian function with FWHM equal to 0.6 mm which corresponds to the characteristic width of the laser beam used for fabrication and tuning of the bottle resonator. Figure 5 compares the input 100 ps pulse (Fig. 5a) and the output pulse for the open and closed bottle unperturbed (Fig. 5b); closed bottle perturbed at its right hand side near the turning point (Fig. 5c–e); and open bottle perturbed at its left hand side (Fig. 5f,g). It is seen that, while the 0.1 Å perturbation of the closed bottle introduces minor distortion of the pulse (Fig. 5c), the distortion becomes significant for the 0.3 Å perturbation (Fig. 5d) and becomes severe for the 0.5 Å perturbation (Fig. 5e). The perturbation profile and 2D plot clarifying the pulse evolution for the last case are shown in Fig. 3c,d. In contrast, the perturbation near the exit of the open bottle resonator up to 2 Å, i.e., ~10% of the effective radius variation (Figs 3e,f and 5f,g) does not cause significant pulse distortion.

## Discussion

Following the original idea of Schrödinger and based on the recent progress in microphotonics, we have introduced and investigated a feasible microscopic optical buffer. It is shown that a few nanometre tuning of the bottle resonator effective radius is sufficient to trap, hold, and release telecommunication optical pulses without distortion over the time period of ten of nanoseconds or longer, while the delay time is limited by the material losses only. The dimensions of this device are determined by the footprint of the SNAP bottle resonator (0.12 mm^2^ for the model considered, which corresponds to 0.009 mm^2^ per a nanosecond delay), while each oscillation of the pulse in this resonator delays light by 3.6 ns (compare with a single-cycle untunable semi-parabolic delay line experimentally demonstrated in^17^, which had the total delay of 2.6 ns and the same footprint). Remarkably, the parabolic profile of the bottle resonator is not the only profile that allows to hold a light pulse with minimal distortion introduced. We show that there exists a wide family of potential wells in which the optical pulse experiences practically no distortion. Exploiting these generalized potentials allows to optimize the performance of miniature optical buffers more efficiently.

We suggest that the exceptionally high precision of 0.1 Å required for the fabrication of the bottle resonator buffer described above is feasible using the advanced SNAP technology. In fact, the precision of 0.7 Å in effective radius variation was experimentally achieved in^21^ by iterations using 0.07 Å increments limited by the resolution of the optical spectrum analyser used. Thus, it is expected that the 0.1 Å precision can be achieved by the straightforward improvement of the SNAP fabrication setup (see Methods).

The tunability of the proposed miniature optical buffer can be achieved utilizing the fibre with a highly nonlinear, electrostrictive, or piezoelectric core^22^,^23^. The local application of a laser field or electric potential to the nonlinear or piezoelectric material positioned inside the fibre allows to deform the fibre, tune its effective radius, and, thus, open and close the bottle resonator. For example, tuning has been recently demonstrated for a silica WGM resonator with a silicon core^22^. The wavelength shift as large as 0.4 nm was introduced by a picosecond laser field and attributed to the Kerr nonlinearity of silicon. This shift corresponds to 5 nm of the effective radius variation for a fibre with the 20 μm radius, which is sufficient to enable the buffering process describe above. Since the required spatial distribution of the switching deformation is smooth (Fig. 3a), the corresponding intensity variation is feasible. The characteristic axial length of the deformable part of the buffer (black lines in Figs 3a and 4a) does not exceed a few millimetres. Consequently, the tunability can be also achieved with a specially designed microscopic piezoelectric transducer glued into a SNAP fibre capillary. The behaviour of a transverse ultrasonic pulse generated by this transducer is generally not adiabatic and is difficult to control dynamically. The relatively slow adiabatic switching can be analysed by measurement of the effective fibre radius variation for the fixed voltage applied to the piezoelectric.

Alternatively, the required nanoscale temporal and spatial variations can be introduced in a fibre segment wholly fabricated of a low loss and highly nonlinear, electrostrictive, and piezoelectric materials (e.g., of silicon^24^ or lithium niobate^25^) for which the SNAP technology can potentially be developed. In this case, the power of the switching laser pulse can be enhanced dramatically if the pulse is resonantly coupled into the fibre WGM^26^. Fabrication of highly nonlinear and electrostrictive fibre segments with the required outstanding uniformity is a fruitful and challenging problem to be addressed in the future.

## Methods

### Derivation of the nonstationary Schrödinger equation

Equation (1) can be derived from Maxwell equations in a way similar to that used in the derivation of the stationary Schrödinger equation which describes propagation of WGM in a SNAP fibre with nanoscale effective radius variation^15^. However, it is more straightforward to derive this equation directly from the stationary Schrödinger equation by the Fourier transform^27^. Assuming that the potential in equation (1) is independent of time, we look for the solution of this equation in the form 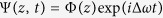, where 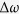 is the frequency variation. After the substitution of this expression into equation (1) we arrive at the known stationary Schrödinger equation for 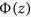
15. The inverse Fourier transform yields equation (1). From^15^, the stationary Schrödinger equation is valid in the vicinity 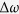 of the resonance frequency much smaller than the free spectral range 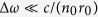. Consequently, the characteristic temporal width 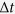 of optical pulses described by equation (1) should satisfy the inequality 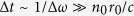. For the characteristic fibre parameters, 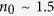 and 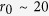
μm^16^,^17^, we have 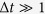 ps. Therefore, the description of nanosecond pulses having 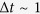 ns by equation (1) is justified.

### Fabrication precision of SNAP structures

It is instructive to discuss the physical meaning of the dramatically high subangstrom precision, which has been achieved in SNAP technology^16^,^21^, and its prospective improvement to 0.1 Å, which is required for the realization of the proposed harmonic optical buffer. The value 0.1 Å is an order of magnitude less than the size of an atom. The definition of such a small measurement precision assumes averaging of the actual surface height variation over the surface dimensions much greater the radiation wavelength. The nanoscale variation of the surface height of SNAP resonators is generally axially asymmetric. For resonators with axial dimensions of several microns and height variation of a few micrometres, the asymmetry may reduce the Q-factor and eventually destroy the resonator^28^,^29^. However, for the SNAP resonators of our interest having the axial length of the order of 100 μm or greater and height of several nanometres, this effect is small^29^. Experimentally, we determine the effective radius variation of the fibre using a microfibre taper connected to the power source and optical spectrum analyser as illustrated in Fig. 2a. The microfibre is translated along the SNAP fibre and the WGM spectrum is measured at sequential points of contacts. In the simplest case of an optical fibre with the slow-varying radius, the radius variation 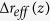 is determined from the shift a WGM resonance 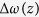 by equation 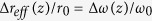
30,31. At characteristic optical frequency of 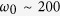 THz (corresponding to the telecommunication wavelength 1.6 μm) the width of the resonance for a silica fibre can be as small as 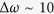
MHz (corresponding to Q-factor ~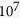). With the same measurement resolution of 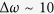 MHz and fibre radius 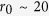
μ m, the measurement precision of the fibre radius variation can be as small as 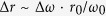 ~ 0.01 Å.

## Additional Information

**How to cite this article**: Sumetsky, M. Microscopic optical buffering in a harmonic potential. *Sci. Rep.* **5**, 18569; doi: 10.1038/srep18569 (2015).

## Supplementary Material

Supplementary Information

## References

[b1] Tucker R. S., Ku P. C. & Chang-Hasnain C. J. Slow-light optical buffers: capabilities and fundamental limitations. J. Lightwave Technol. 23, 4046–4066 (2005).

[b2] Khurgin J. B. Slow light in various media: a tutorial. Adv. Opt. Photon. 2, 287–318 (2010).

[b3] Xia F. N., Sekaric L. & Vlasov Y. Ultracompact optical buffers on a silicon chip. Nat. Photon. 1, 65–71 (2007).

[b4] Baba T. Slow light in photonic crystals. Nat. Photon. 2, 465–473 (2008).

[b5] Notomi M. Manipulating light with strongly modulated photonic crystals. Rep. Prog. Phys. 73, 096501 (2010).

[b6] Yanik M. F. & Fan Y. Stopping light all optically. Phys. Rev. Lett. 92, 083901 (2004). 14995773 10.1103/PhysRevLett.92.083901

[b7] Miller D. A. B. Fundamental Limit to Linear One-Dimensional Slow Light Structures. Phys. Rev. Lett. 99, 203903 (2007). 18233141 10.1103/PhysRevLett.99.203903

[b8] Mookherjea S., Park J. S., Yang S.-H. & Bandaru P. R. Localization in silicon nanophotonic slow-light waveguides. Nat. Photon. 2, 90–93 (2008).

[b9] Burmeister E. F., Blumenthal D. J. & Bowers J. E. A comparison of optical buffering technologies. Opt. Switching and Netw. 5, 10–18 (2008).

[b10] Schrödinger E. Der stetige Übergang von der Mikro- zur Makromechanik. *Naturwissenschaften* **14**, 664 (1926); translated and reprinted as “The continuous transition from micro- to macro mechanics,” in Collected papers on wave mechanics (Chelsea Publishing, New York, 1982) pp. 41–44

[b11] Robinett R. W. Quantum wave packet revivals. Phys. Rep. 392, 1–119 (2004).

[b12] Poladian L. Phys. Rev. E 48, 4758–4767 (1993). 10.1103/physreve.48.4758 9961159

[b13] Istrate E. & Sargent E. H. Photonic crystal heterostructures and interfaces. Rev. Mod. Phys. 78, 455–481 (2006).

[b14] Sumetsky M. CROW bottles. Opt. Lett. 39, 1913–1916 (2014). 24686637 10.1364/OL.39.001913

[b15] Sumetsky M. & Fini J. Surface nanoscale axial photonics. Opt. Express 19, 26470–26485 (2011). 22274232 10.1364/OE.19.026470

[b16] Sumetsky M. Nanophotonics of optical fibers. Nanophotonics 2, 393–406 (2013).

[b17] Sumetsky M. Delay of light in an optical bottle resonator with nanoscale radius variation: dispersionless, broadband, and low loss. Phys. Rev. Lett. 111, 163901 (2013). 24182267 10.1103/PhysRevLett.111.163901

[b18] Landau L. D. & Lifshitz E. M. Quantum Mechanics (Pergamon, New York, 1958).

[b19] Frieschy O. M., Marzoliz I. & Schleich W. P. Quantum carpets woven by Wigner functions. New Journ. Phys. 2, 4.1–4.11 (2000).

[b20] Yanik M. F. & Fan S. Stopping and storing light coherently. Phys. Rev. **A** 71, 013803 (2005).

[b21] Sumetsky M. & Dulashko Y. SNAP: Fabrication of long coupled microresonator chains with sub-angstrom precision. Opt. Express 20, 27896–27901 (2012). 23262734 10.1364/OE.20.027896

[b22] Suhailin F. H., Healy N., Franz Y., Sumetsky M., Ballato J., Dibbs A. N., Gibson U. J. & Peacock A. C. Kerr nonlinear switching in a hybrid silica silicon microspherical resonator. Opt. Express. 23, 17263–17268 (2015). 26191735 10.1364/OE.23.017263

[b23] Homan D., Kaur G., Pickrell G., Scott B. & Hill C. Electronic and magnetic fibers. Mat. Lett. 133, 135–138 (2014).

[b24] Vukovic N., Healy N., Suhailin F. H., Mehta P., Day T. D., Badding J. V. & Peacock A. C. Ultrafast optical control using the Kerr nonlinearity in hydrogenated amorphous silicon microcylindrical resonators. Sci. Rep. 3, 2885–2889 (2013). 24097126 10.1038/srep02885 PMC3791441

[b25] Yin S. Lithium Niobate fibers and waveguides: fabrications and applications. Proc. IEEE 87, 1962–1974 (1999).

[b26] Pöllinger M. & Rauschenbeutel A. All-optical signal processing at ultra-low powers in bottle microresonators using the Kerr effect. Opt. Express 18, 17764–17775 (2010). 20721164 10.1364/OE.18.017764

[b27] Agrawal G. P. Nonlinear fiber optics (Academic Press, 2013).

[b28] Ferdous F., Demchenko A. A., Vyatchanin S. P., Matsko A. B. & Maleki L. Microcavity morphology optimization. Phys. Rev. **A** 90, 033826 (2014).

[b29] Kochkurov L. A. & Sumetsky M. Nanobump microresonator. Opt. Lett. 40, 1430–1432 (2015). 25831350 10.1364/OL.40.001430

[b30] Birks T.A., Knight J. C. & Dimmick T. E. High-resolution measurement of the fiber diameter variations using whispering gallery modes and no optical alignment. IEEE Photon. Technol. Lett. 12, 182–183 (2000).

[b31] Sumetsky M. & Dulashko Y. Radius variation of optical fibers with angstrom accuracy. Opt. Lett. 35, 4006–4008 (2010). 21124594 10.1364/OL.35.004006

