# Supplementary material for: Microscopic optical buffering in a harmonic potential
Source: Sci Rep. 2015 Dec 22;5:18569. doi: 10.1038/srep18569 (PMC4686970; doi:10.1038/srep18569)
Supplement: Supplementary Information [file srep18569-s1.pdf]

Microscopic optical buffering in a harmonic potential

M. Sumetsky

Aston Institute of Photonic Technologies, Aston University, Birmingham B4 7ET, UK

m.sumetsky@aston.ac.uk

Supplementary Note

In classical mechanics, the potential wells with equidistant spectrum correspond to those having the period of oscillations independent of amplitude. There exist a wide range of such potentials, while a quadratic potential is their simplest representative. Consider a classical particle with energy E and mass m oscillating in a potential well $V(z)$ between turning points $z_1(E)$ and $z_2(E)$. The period of oscillations $T(E)$ is defined by the integral [1]:

$$T(E)=\sqrt{2m}\int_{z_1(E)}^{z_2(E)}\frac{dz}{\sqrt{E-V(z)}} \tag{A.1}$$

This equation can be also considered as an integral equation (Abel integral equation [2]) which determines potential $V(z)$ for the given dependence of the period on the energy $T(E)$. Analytical solution of this equation allows to express the inverse function $z(V)$ through $T(E)$ [1, 2]. Since $V(z)$ is a potential well, we assume that the function $z(V)$ is two-valued and can be separated into two monotonic branches $z^{(1)}(V)$ and $z^{(2)}(V)$ equal to each other at a common minimum z_0, where $V(z_0)=V_0$ and $z^{(1)}(V_0)=z^{(2)}(V_0)=z_0$. Then solution of the integral equation (A.1) yields [1]:

$$z^{(2)}(V)-z^{(1)}(V)=\frac{1}{\pi\sqrt{2m}}\int_{V_0}^{V}\frac{T(E)dE}{\sqrt{V-E}} \tag{A.2}$$

Here we are searching for a harmonic (but not necessarily quadratic) potential, i.e., the potential having the oscillation period independent of energy, $T(E)\equiv T_0$. In this case, equation (A.2) is simplified and yields the family of harmonic potentials defined by the algebraic equation:

$$z^{(2)}(V) - z^{(1)}(V) = C\left(\sqrt{V - V_0}\right) \tag{A.3}$$

where $C = (2/m)^{1/2} T_0 / \pi$. In this equation one branch of the potential, e.g., $z^{(1)}(V)$, can be an arbitrary monotonic function, while the other branch, $z^{(2)}(V)$, is expressed through $z^{(1)}(V)$ from equation (A.3).

References

[1] L. D. Landau and E. M. Lifshitz, Mechanics (Pergamon, New York, 1960).

[2] R. Gorenflo and S. Vessella, Abel Integral Equations, Vol. 1461 of Lecture Notes in Mathematics Series, A. Dold, B. Eckmann, and F. Takens, eds. (Springer-Verlag, Berlin, 1991).
